# Automatic design of decision-tree induction algorithms tailored to flexible-receptor docking data

**DOI:** 10.1186/1471-2105-13-310

**Published:** 2012-11-21

**Authors:** Rodrigo C Barros, Ana T Winck, Karina S Machado, Márcio P Basgalupp, André CPLF de Carvalho, Duncan D Ruiz, Osmar Norberto de Souza

**Affiliations:** 1 University of São Paulo, São Carlos, Brazil; 2 Federal University of Santa Maria, Santa Maria, Brazil; 3 Federal University of Rio Grande, Rio Grande, Brazil; 4 Federal University of São Paulo, São José dos Campos, Brazil; 5 Pontifical Catholic University of Rio Grande do Sul, Porto Alegre, Brazil

## Abstract

**Background:**

This paper addresses the prediction of the free energy of binding of a drug candidate with enzyme *InhA* associated with *Mycobacterium tuberculosis*. This problem is found within *rational drug design*, where interactions between drug candidates and target proteins are verified through molecular docking simulations. In this application, it is important not only to correctly predict the free energy of binding, but also to provide a comprehensible model that could be validated by a domain specialist. Decision-tree induction algorithms have been successfully used in drug-design related applications, specially considering that decision trees are simple to understand, interpret, and validate. There are several decision-tree induction algorithms available for general-use, but each one has a bias that makes it more suitable for a particular data distribution. In this article, we propose and investigate the automatic design of decision-tree induction algorithms tailored to particular drug-enzyme binding data sets. We investigate the performance of our new method for evaluating binding conformations of different drug candidates to *InhA*, and we analyze our findings with respect to decision tree accuracy, comprehensibility, and biological relevance.

**Results:**

The empirical analysis indicates that our method is capable of automatically generating decision-tree induction algorithms that significantly outperform the traditional C4.5 algorithm with respect to both accuracy and comprehensibility. In addition, we provide the biological interpretation of the rules generated by our approach, reinforcing the importance of comprehensible predictive models in this particular bioinformatics application.

**Conclusions:**

We conclude that automatically designing a decision-tree algorithm tailored to molecular docking data is a promising alternative for the prediction of the free energy from the binding of a drug candidate with a flexible-receptor.

## Background

The pharmaceutical industry is under increasing pressure to continuously deliver new drugs to the market [1]. Since the costs involved in the development of new drugs have exceeded one billion dollars, *Rational Drug Design* (RDD) has become an emerging technology for cost reduction and fast development of new drugs [2].

Interaction between drug candidates (ligands) and target proteins (receptors) through molecular docking simulations is the computational basis of RDD. Given a receptor, molecular docking simulations sample a large number of orientations and conformations of a ligand inside the protein bibding site. The simulations also evaluate the Free Energy of Binding (FEB) and rank the orientations/conformations according to their FEB scores [3].

Nowadays, the majority of molecular docking algorithms only consider the ligand as flexible whereas the receptor remains rigid, due to the computational cost when considering its flexibility. Conversely, biological macromolecules, like protein receptors, are intrinsically flexible in their cellular environment, considering that the receptor may modify its shape upon ligand binding, moulding itself to be complementary to its ligand. This increases favorable contacts and reduces adverse interactions, which in turn minimizes the total FEB [4]. Therefore, it is important to consider the receptor flexibility during molecular docking.

**Figure 1:**
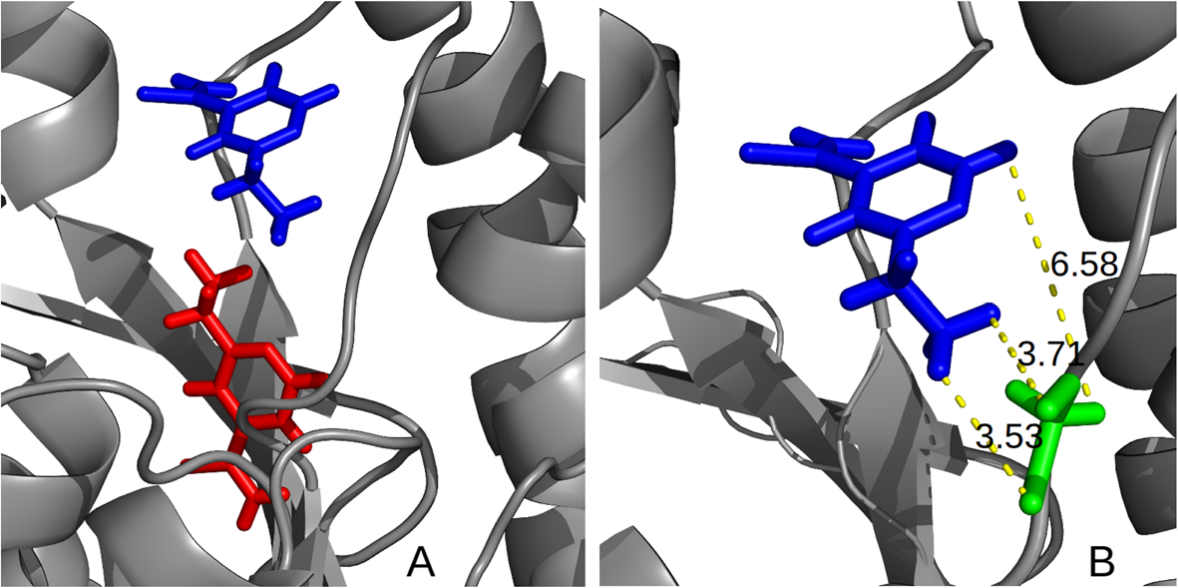
**Molecular docking simulation.** Figure 1 is divided as follows: (**A**) shows an example of a docking simulation from *InhA*, where the protein in ribbon is depicted in gray, the *ETH* ligand in its initial position is highlighted in red, and the final position of *ETH* after a molecular docking experiment is highlighted in blue; (**B**) presents an example of the distances between the *ETH* ligand and the receptor residue *GLY95* (Glycine 95).

Among all available methodologies to explicitly include the receptor flexibility in molecular docking simulations, a possible alternative is to select a series of different conformations derived from a molecular dynamics (MD) simulation of the target receptor [5]. We name this type of receptor representation *fully flexible-receptor* (FFR) model [6,7], and we investigate this methodology with target receptor *InhA* enzyme from *Mycobacterium tuberculosis*[8] (Mtb), which was modeled as a set of 3,100 snapshots derived from a 3.1 ns MD simulation trajectory [9]. For that, we generated molecular docking data sets with data from docking simulations of *FFR-InhA*[10] to six different ligands: *nicotinamide adenine dinucleotide* (*NADH*) [8], *triclosan* (*TCL*) [11], *pentacyano(isoniazid)ferrate(II)* (*PIF*) [12], *ethionamide* (*ETH*) [13], *Isoniazid* (*INH*) [14], and *Triclosan derivative 20* (*JPM*) [15]. Explicitly including the receptor flexibility in docking simulations is computationally demanding and generates large amounts of data, which need to be analyzed and interpreted. The concept of molecular docking is better illustrated in Figure 1.

Decision-tree induction algorithms have been successfully used in drug-design related applications [16-19]. One of the main advantages of these algorithms when compared to other machine learning techniques (*e.g.*, SVMs and Neural Networks) is that decision trees are simple to understand, interpret and validate. Thus, domain specialists (*e.g.,* biologists, physicians, chemists) can easily verify whether the data present interesting patterns, increasing their confidence in making predictions and creating new hypotheses. Several decision-tree induction algorithms have been proposed for general-use, but each has a bias that makes it more suitable for a particular data distribution. Hence, a common choice for decision-tree applications is to employ state-of-the-art decision-tree induction algorithm C4.5 [20], regardless of the fact that it was not tailored to the biological domain of interest.

In this article, we investigate a new data mining approach that automatically generates new decision-tree algorithms tailored to a specific domain. We employ this new approach for analyzing data from fully flexible-receptor molecular docking experiments, looking for receptor snapshots to which a particular ligand binds more favorably. With the resulting induced models from these automatically-designed algorithms, we expect that the inferred knowledge will help us to point out which of the conformations that were generated by the fully flexible-receptor model are more promising to future docking experiments. This, in turn, allows a reduction of the flexible-receptor model dimensionality and permit faster docking simulations of flexible receptors [6]. We analyze whether the decision trees generated by the automatically-designed algorithms have higher predictive accuracy and are more comprehensible than decision trees generated by state-of-the-art decision-tree induction algorithm, C4.5 [20]. In addition, we interpret and validate our findings with the help of a domain specialist.

## Method

In this section, we describe the proposed method for automatically generating decision-tree induction algorithms tailored to flexible-receptor molecular docking data, namely *Hyper-heuristic Evolutionary Algorithm for automatically Designing Decision-Tree algorithms* (HEAD-DT) [21]. First, we briefly introduce decision trees, and the importance of generating comprehensible models.

### Decision trees background

Automatically generating rules in the form of decision trees has been a key active research topic in the development of data exploration techniques [22]. Disciplines such as engineering (pattern recognition), statistics, decision theory, and more recently artificial intelligence (machine learning) have a large number of works dedicated to the generation and application of decision trees.

Formally, a basic top-down decision-tree induction algorithm can be recursively defined in only two steps, in the so-called *Hunt’s algorithm*. Let **X**_**t**_be a set of training instances associated with node *t* and *y*={*y*_1_*y*_2_,…,*y*_*k*_} be the set of class labels in a *k*-class problem [23]: 

1) if all the instances from **X**_**t**_belong to the same class *y*_*t*_then *t* is a leaf node labeled as *y*_*t*_;

2) if **X**_**t**_contains instances that belong to more than one class, an attribute test condition is selected to partition the instances into subsets. A child node is created for each outcome of the test and the instances in **X**_**t**_are distributed to the children based on the outcomes. Recursively apply the algorithm to each child.

This simplified algorithm is is the basis for all current top-down decision tree induction algorithm. Nevertheless, its assumptions are too stringent for practical use. For instance, it would only work if every combination of attribute values is present in the training data, and if the training data is inconsistency-free (each combination has a unique class label). Hunt’s algorithm was improved in many ways. Its **stopping criterion**, for example, as expressed in *step 1*, requires all leaf nodes to be pure (*i.e.*, belonging to the same class). In most practical cases, this constraint leads to enormous decision trees, which tend to suffer from *overfitting*. Possible solutions to overcome this problem is prematurely stopping the tree growth when a minimum level of impurity is reached, or performing a **pruning** step after the tree has been fully grown. Another design issue is how to properly select the **split** test to partition the instances into smaller subsets. In Hunt’s original approach, a cost-driven function was responsible for partitioning the tree. Subsequent algorithms such as ID3 [24] and C4.5 [20] make use of information-theory based functions for partitioning nodes in purer subsets. Finally, dealing with **missing values** is also a major design issue one has to face when developing a new decision-tree induction algorithm.

Alternatives to the top-down approach were proposed in the last decades, such as bottom-up induction [25], evolutionary induction [26-30], and ensemble of trees [31]. Notwithstanding, no strategy has been more successful in generating accurate and comprehensible decision trees with low computational effort than the greedy top-down induction strategy. Due to its popularity, a large number of approaches have been proposed for each one of the *design components* of top-down decision-tree induction algorithms. Considering that the *manual* improvement of decision-tree design components has been carried out for the past 40 years, we believe that *automatically* designing decision-tree induction algorithms could provide a faster, less-tedious — and equally effective — strategy for improving decision-tree algorithms. Hence, we propose in this work to automatically generate new and effective decision-tree algorithms tailored to the flexible-receptor molecular docking data.

We recall that decision-tree induction algorithms are widely-used for knowledge discovery and pattern recognition tasks, due to their advantage of producing a *comprehensible* classification model. Notwithstanding, these algorithms are usually underestimated in bioinformatics, given that researchers tend to prefer methods such as support vector machines or neural networks [32-34]. These methods are usually very effective in terms of predictive accuracy, but they are “black box” methods, providing little biologically-meaningful explanation for their prediction, giving few new insight about the data or the application domain to users [35]. In many bioinformatics applications, however, the discovered model should be interpreted and validated in the context of current biological knowledge.

### HEAD-DT

**Figure 2:**
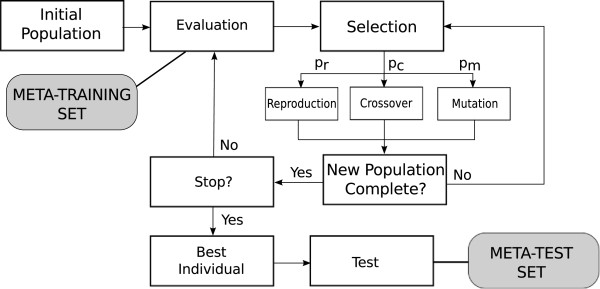
**HEAD-DT evolutionary scheme.** Figure 2 presents the evolutionary scheme followed by HEAD-DT. A random initial population of individuals (decision-tree algorithms) is created and evaluated according to the performance of their corresponding trees in a meta-training set. Then, a selection procedure is responsible for choosing individuals that will undergo breeding operations. After a new population is complete, it is once again evaluated and the process continues until a maximum number of generations is reached. The best decision-tree induction algorithm is then executed over a meta-test set, which estimates its performance in unseen data.

HEAD-DT is a hyper-heuristic algorithm able to automatically design top-down decision-tree algorithms [21,36]. Hyper-heuristics can automatically generate new heuristics suited to a given problem or class of problems. This is carried out by combining, through an evolutionary algorithm, components or building-blocks of human designed heuristics [37]. HEAD-DT is a regular generational evolutionary algorithm, in which individuals are collections of building blocks of decision-tree algorithms. Figure 2 illustrates the evolutionary scheme followed by HEAD-DT. Each individual is encoded as an integer string and each gene has a different range of supported values. We divided the genes into four categories, representing the major building blocks (design components) of a decision-tree algorithm: split genes, stopping criteria genes, pruning genes, and missing values genes. We detail each category next.

#### Split genes

These genes are used for selecting the attribute to split the data in the current node of the decision tree. A decision rule based on the selected attribute is thus generated, and the input data is filtered according to the outcomes of this rule. This process continues recursively. We used two genes to model the split component of a decision-tree algorithm. The first gene, with an integer value, indexes one of the 15 splitting criteria implemented: information gain [24], Gini index [38], mutual information [39], G statistics [40], Mantaras criterion [41], hypergeometric distribution [42], Chandra-Varghese criterion [43], DCSM [44], *χ*^2^[45], mean posterior improvement [46], normalized gain [47], orthogonal criterion [48], twoing [38], CAIR [49] and gain ratio [20]. The second gene, with a binary value, represents the split component of a decision-tree algorithm, indicating whether the splits of a decision tree will be necessarily binary or multi-edged. In a binary tree, every split has only two outcomes (edges). Thus, nominal attributes with many categories have to be divided into two subsets, each representing an aggregation over several categories. In a multi-edge tree, nominal attributes are divided according to their number of categories, *i.e.*, one edge for each category. In both cases, numeric attributes always partition the tree into two subsets (*att*≤ *threshold*, *att*>*threshold*).

#### Stopping criteria genes

The second category of genes concerns the stopping criteria component of decision-tree induction algorithms. The top-down induction of a decision tree is recursive and it continues until a stopping criterion is satisfied. We implemented the following stopping criteria: 

1) Reaching class homogeneity — when all instances that reach a given node belong to the same class, there is no reason to split this node any further. This strategy can be combined with any of the following strategies;

2) Reaching the maximum tree depth — a parameter *tree depth* can be specified to avoid deep trees. We have fixed its range in the interval [2,10] levels;

3) Reaching the minimum number of instances for a non-terminal node — a parameter *minimum number of instances for a non-terminal node* can be specified to avoid (or at least alleviate) the data fragmentation problem in decision trees. Range: [1,20] instances;

4) Reaching the minimum percentage of instances for a non-terminal node — same as before, but instead of the current number of instances, we set the minimum percentage of instances. Its range is [1*%*,10*%*] of the total number of instances;

5) Reaching an accuracy threshold within a node — a parameter *accuracy reached* can be specified to stop the growth of the tree when the accuracy within a node (majority of instances) reaches a given threshold. Possible values are {70*%*,75*%*,80*%*,85*%*,90*%*,95*%*,99*%*}.

The first of the stopping criteria genes selects one of the five different strategies for stopping the tree growth. The second gene dynamically adjusts a value within the range [0,100] to the corresponding strategy. For example, if the strategy selected by the first gene is *reaching the maximum tree depth*, the following mapping function is executed: *result*=(*value* mod 9) + 2. This function maps from [0,100] to [2,10], which is what was defined as the range of this strategy.

#### Pruning genes

Pruning is usually performed in decision trees for enhancing tree comprehensibility by reducing its size while maintaining (or even improving) accuracy. We implemented the following well-known pruning strategies: i) reduced-error pruning; ii) pessimistic error pruning; iii) minimum error pruning; iv) cost-complexity pruning; and v) error-based pruning. 

1) Reduced-error pruning (REP) is a conceptually simple strategy proposed by Quinlan [50]. It uses a pruning set to evaluate the goodness of a given subtree from *T*. The idea is to evaluate each non-terminal node *t* with regard to the classification error in the pruning set. If such an error decreases when we replace the subtree *T*^(*t*)^rooted on *t* by a leaf node, then *T*^(*t*)^must be pruned. Quinlan imposes a constraint: a node *t* cannot be pruned if it contains a subtree that yields a lower classification error in the pruning set. The practical consequence of this constraint is that REP should be performed in a bottom-up fashion.

2) Pessimistic error pruning (PEP) [50] uses the training set for both growing and pruning the tree. The apparent error rate is optimistically biased and cannot be used to decide whether pruning should be performed or not. Quinlan thus proposes adjusting the apparent error according to the continuity correction for the binomial distribution in order to provide a more realistic error rate. PEP is computed in a top-down fashion, and if a given node *t* is pruned, its descendants are not examined, which makes this pruning strategy efficient in terms of computational effort.

3) Minimum error pruning (MEP) [51] is a bottom-up approach that seeks to minimize the *expected error rate* for unseen cases. It uses an ad-hoc parameter *m* for controlling the level of pruning. Usually, the higher the value of *m*, the more severe the pruning. Cestnik and Bratko [51] suggest that a domain expert should set *m* according to the level of noise in the data. Alternatively, a set of trees pruned with different values of *m* could be offered to the domain expert, so he/she can choose the best one according to his/her experience.

4) Cost-complexity pruning (CCP) is the post-pruning strategy of the CART system [38]. It consists of two steps: (i) generate a sequence of increasingly smaller trees, beginning with *T* and ending with the root node of *T*, by successively pruning the subtree yielding the lowest *cost complexity*, in a bottom-up fashion; (ii) choose the best tree among the sequence based on its relative size and accuracy (either on a pruning set, or provided by a cross-validation). The idea within step (i) is that pruned tree *T*_*i* + 1_is obtained by pruning the subtrees that show the lowest increase in the apparent error (error in the training set) per pruned leaf. Regarding step (ii), CCP chooses the smallest tree whose error (either on the pruning set or on cross-validation) is not more than one standard error greater than the lowest error observed in the sequence of trees.

5) Error-based pruning (EBP) was proposed by Quinlan and it is implemented as the default pruning strategy of C4.5 [20]. It is an improvement over PEP, based on a far more pessimistic estimate of the expected error. Unlike PEP, EBP performs a bottom-up search, and it carries out not only the replacement of non-terminal nodes by leaves but also *grafting* of subtree *T*^(*t*)^onto the place of parent *t*. For deciding whether to replace a non-terminal node by a leaf (subtree replacement), to graft a subtree onto the place of its parent (subtree raising) or not to prune at all, a pessimistic estimate of the expected error is calculated by using an upper confidence bound.

We designed two genes in HEAD-DT for pruning. The first gene indexes one of the five approaches for pruning a DT (and also the option of not pruning at all). The second gene is in the range [0,100] and its value is dynamically mapped by a function, according to the pruning method selected (similar to the second stopping criteria gene). For REP, the parameter is the percentage of training data to be used in the pruning set (varying within the interval [10*%*,50*%*]). For PEP, the parameter is the number of standard errors (SEs) to adjust the apparent error, in the set {0.5,1,1.5,2}. For MEP, the parameter *m* may range within [0,100]. For CCP, there are two parameters: the number of SEs (in the same range than PEP) and the pruning set size (in the same range than REP). Finally, for EBP, the parameter *CF* may vary within [1*%*,50*%*].

#### Missing values genes

Handling missing values is an important issue in decision-tree induction and its use for classification. We designed three genes for dealing with missing values in distinct scenarios: (i) during split evaluation; (ii) during instance distribution; and (iii) during classification, as follows.

For the split criterion evaluation of node *t* based on attribute *a*_*i*_, we implemented the following strategies: 1) ignore all instances whose value of *a*_*i*_ is missing; 2) imputation of missing values with either the mode (nominal attributes) or the mean (numeric attributes) of all instances in *t*; 3) weight the splitting criterion value (calculated in node *t* with regard to *a*_*i*_) by the proportion of missing values; 4) imputation of missing values with either the mode (nominal attributes) or the mean (numeric attributes) of all instances in *t* whose class attribute is the same of the instance whose *a*_*i*_ value is being imputed.

For deciding which child node training instance *x*_*j*_should go to, considering a split in node *t* over *a*_*i*_, we adopted the following options: 1) ignore instance *x*_*j*_; 2) treat instance *x*_*j*_ as if it has the most common value of *a*_*i*_, regardless of the class; 3) treat instance *x*_*j*_ as if it has the most common value of *a*_*i*_considering the instances that belong to the same class than *x*_*j*_; 4) assign instance *x*_*j*_ to all partitions; 5) assign instance *x*_*j*_to the partition with the largest number of instances; 6) weight instance *x*_*j*_ according to the partition probability; 7) assign instance *x*_*j*_ to the most probable partition, considering the class of *x*_*j*_.

Finally, for classifying a new test instance *x*_*j*_, considering a split in node *t* over *a*_*i*_, we used the strategies: 1) explore all branches of *t* combining the results; 2) take the route to the most probable partition (largest subset); 3) stop the classification process and assign instance *x*_*j*_ to the majority class of node *t*.

#### Evolution and fitness evaluation

The evolution of individuals in HEAD-DT follows the scheme presented in Figure 2. The 9-gene linear genome of an individual in HEAD-DT is comprised of the building blocks described in the earlier sections: *[split criterion, split type, stopping criterion, stopping parameter, pruning strategy, pruning parameter, mv split, mv distribution, mv classification].* One possible individual encoded by that linear string is [4,1,2,77,3,91,2,5,1], which accounts for the following algorithm: 

1)Recursively split nodes with the G statistics criterion;

2)Create one edge for each category in a nominal split;

3)Perform step 1 until class-homogeneity or the maximum tree depth of 7 levels ((77 mod 9) + 2) is reached;

4)Perform MEP pruning with m = 91;

5)When dealing with missing values:

5.1)Impute missing values with mode/mean during split calculation;

5.2)Distribute missing-valued instances to the partition with the largest number of instances;

5.3)For classifying an instance with missing values, explore all branches and combine the results.

**Figure 3:**
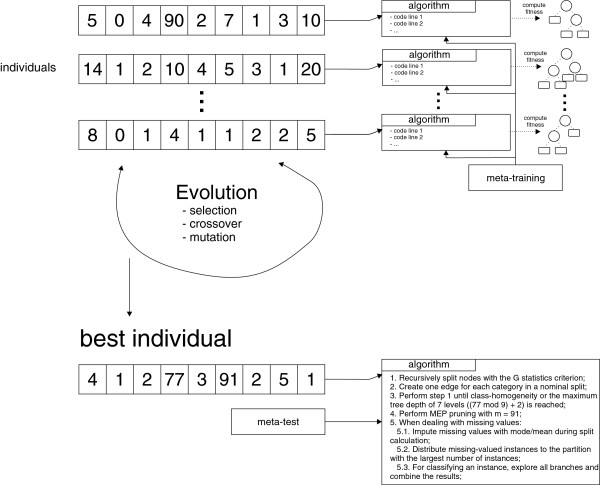
**HEAD-DT in action.** Figure 3 presents an illustration of HEAD-DT — a set of linear-genome individuals are decoded into algorithms and executed over the meta-training set. The performance of these individuals is measured and the evolutionary cycle starts with typical operations such as selection, crossover, and mutation. At the end of the cycle, the best (fitness-wise) individual is selected for inducing decision trees from the meta-test set.

Figure 3 presents an example of how linear genomes are decoded into algorithms, and how they participate of the evolutionary cycle. The first step of HEAD-DT is the generation of the initial population, in which a population of 100 individuals is randomly generated (random number generation within the genes acceptable range of values). Then, the individuals participate in a pairwise tournament selection procedure for defining those that will undergo genetic operators. Individuals may participate in either one-point crossover (80% probability), random uniform gene mutation (15% probability), or reproduction (5% probability), the three mutually-exclusive genetic operators employed in HEAD-DT. In addition, HEAD-DT employs an elitism strategy, in which the best 5 individuals are kept from one generation to the next.

During fitness evaluation, a *meta-training set* is used for assessing the quality of each individual throughout evolution. The *meta-test set* is used to assess the quality of the decision-tree induction algorithm evolved by HEAD-DT (the best individual in Figure 2). There are two distinct approaches for dealing with the meta-training and test sets: (i) evolving a decision-tree induction algorithm tailored to one specific data set; and (ii) evolving a single decision-tree induction algorithm to be employed in multiple data sets. In the first case, we have a specific data set to which we want to design a decision-tree induction algorithm. In the second case, we have one (or several) data set(s) comprising the meta-training set, and multiple data sets comprising the meta-test set.

We perform experiments with both approaches previously described. In the first set of experiments, we evolved a decision-tree induction algorithm tailored to each molecular docking data set. In the second set, we evolved a single decision-tree induction algorithm, using only one data set, and applied this algorithm to all data sets. In the first scenario, we use the classification accuracy of a validation set (25% the size of the training set) to evolve the individuals (decision-tree algorithms) of HEAD-DT. In the second scenario, we use the classification accuracy obtained by performing 10-fold cross-validation in the meta-training set as our fitness function.

## Results

The hypothesis we try to confirm in this paper is that automatically-designed decision-tree induction algorithms can be more effective than human-designed, general-use decision-tree induction algorithms for solving problems of a particular domain. More specifically, we investigate the problem of RDD through flexible-receptor molecular docking simulations.

One way to evaluate a molecular docking simulation (*e.g.AutoDock*) is by examining the resulting FEB value: the smaller the FEB value, the better the binding of the ligand into the receptor binding pocket. *AutoDock* is a suit of programs used to predict the bound conformations of a ligand to a receptor, which applies a technique that combines an algorithm of conformation-searching with a rapid grid-based method of energy evaluation [52]. This grid-based method is performed by the module of *AutoDock* called *AutoGrid*. It pre-calculates a three-dimensional energy-based grid of interactions of various atom types. *AutoGrid* generates a a grid map for each atom in the ligand considering a probe atom that visits each grid point. The interaction energy between the ligand and the probe atom is then calculated and stored. To estimate the final FEB value, *AutoDock* applies an empirical binding free energy function where the molecular mechanism-based and empirical terms are multiplied by coefficients obtained by linear regression analysis [52].

An important feature related to FEB is the Euclidean distance (measured in Angstrom, Å) between atoms in the receptor’s residues and ligands. Thus, for each receptor-amino acid residue, we calculate the distances between theirs and the ligand atoms. For all calculated distances, we only consider the shortest distance for each receptor residue. Therefore, each receptor residue may be seen as a predictive attribute in a data mining problem. Given that the *InhA* receptor contains 268 amino acid residues, each docking data set has 268 predictive attributes plus the FEB class, which is the attribute we are interested in predicting [53]. Each instance in a molecular docking data set is a receptor snapshot. We produced a distinct data set for each of the six previously mentioned ligands (see Table 1).

**Table 1:** Summary of the data sets

Ligand	# Instances	# Attributes	# Classes	Class Distribution
NADH	2,823	268 (97)	5	205-1020-374-903-321
ETH	3,043	268 (108)	5	160-512-2131-226-14
PIF	3,042	268 (106)	5	7-223-2616-173-23
TCL	2,837	268 (78)	5	19-158-1866-645-149
INH	2,953	268 (89)	5	12-260-2420-175-86
JPM	2,786	268 (80)	5	5-201-1835-323-421

Table 1 presents the summary of the flexible-receptor docking data sets used in the experiments. Number os instances represents the total of valid docking results, out of 3,100. Number of attributes shows the total of selected attributes, in parenthesis, from the initial 268. Class distributions are regarding the five FEB classes, respectively: excellent, good, regular, weak, negligible.

Since FEB is a continuous variable, we employ a discretization technique detailed in [54], which divides FEB in five levels of binding quality. Such technique makes use of the mode and standard deviation of FEB, dividing its sorted values into intervals where border values of the distribution fit each instance into its proper class. The border values are shown in Equation 1, where *M*_*o*_ and *σ* represents the mode and standard deviation values of the *FEB* distribution.

(1) $$\text{Class}=\left\{\;\begin{array}{cccccc} \text{Excelent} & \;\;\text{if}\;\;M_{o}-2*\sigma & >\;\; & \text{FEB}\\ \text{Good} & \;\;\text{if}\;\;M_{o}-\sigma & >\;\; & \text{FEB} & \geq \;\; & M_{o}-2*\sigma \\ \text{Regular} & \;\;\text{if}\;\;M_{o}+\sigma & >\;\; & \text{FEB} & \geq \;\; & M_{o}-\sigma \\ \text{Weak} & \;\;\text{if}\;\;M_{o}+2*\sigma & >\;\; & \text{FEB} & \geq \;\; & M_{o}+\sigma \\ \text{Negligible} & \;\;\text{if} & \;\; & \text{FEB} & >\;\; & M_{o}+2*\sigma \end{array}\right)$$

Moreover, we perform attribute selection to reduce the 268-dimensional data sets using the following procedure: we remove all attributes (residues) whose shortest distance to the ligand is larger than 5 Å (distances larger than 5 Å do not establish a meaningful contact between receptor and ligand atoms). The number of disjointed attributes is within parentheses in Table 1.

We perform two different kinds of experiments — one for each fitness strategy previously detailed. In the first experiment, the meta-training set is comprised of the *NADH* data set (which is the natural ligand for receptor *InhA*). The remaining five data sets (meta-test set) are then used for assessing the performance of the decision-tree algorithm that was tailored to the *NADH* data. For each of the five data sets, a 10-fold cross-validation procedure is performed. In the second experiment, HEAD-DT automatically designs a decision-tree algorithm tailored to each of the six data sets. Thus, each data set is divided in training and test sets, in a 10-fold cross-validation procedure, and then HEAD-DT designs an algorithm tailored to each of the training folds. We analyze the average performance of HEAD-DT in the 10-folds, considering both *test accuracy* and *tree size* (total number of nodes) of the corresponding decision trees.

In order to provide some reassurance about the validity and non-randomness of the obtained results, we present the results of the *corrected resampled t-test statistic*, following the approach proposed by Nadeau and Bengio [55]. Considering that the standard t-test has a high *Type-I* error when used in conjunction with random subsampling, Nadeau and Bengio [55] observe that this is due to an underestimation of the variance because the samples are not independent (*i.e.*, the different training and test sets overlap). Consequently, they propose to correct the variance estimate by taking this dependency into account. Let *a*_*j*_ and *b*_*j*_be the accuracy of algorithms *A* and *B* respectively, measured on run *j* (1≤*j*≤*k*). Assume that in each run, *n*_1_ instances are used for training and the remaining *n*_2_instances for testing. Let *dif*_*j*_be the difference *dif*_*j*_=*a*_*j*_−*b*_*j*_, and $\hat{\mu }$ and ${\hat{\sigma }}^{2}$ the estimates of mean and variance of the *k* differences. The statistic of the *corrected resampled t-test* is:

(2) $$t=\frac{\frac{1}{k}\overset{k}{\underset{j=1}{\sum }}\text{di}f_{j}}{\sqrt{\left(\frac{1}{k}+\frac{n_{2}}{n_{1}}\right)\times {\hat{\sigma }}^{2}}}.$$

Given that we employ a 10-fold cross-validation procedure, *k*=10 and (*n*_2_/*n*_1_)=(1/9). To reject the null hypothesis of equal performances between the algorithms, the value of *t* is tested regarding the Student-*t* distribution, with *k*−1 degrees of freedom and *α* is adjusted to (1−*α*)/2, *i.e.*, *t*_*k*−1,1−*α*/2_. Considering *α*=0.95 and 9 degrees of freedom, *i.e.*, *t*_9,0.025_=2.26216, the null hypothesis is rejected if *t*>2.26216.

### Experiment 1 — An evolved decision-tree algorithm tailored to the *NADH* data set

Table 2 shows the classification accuracy and tree size of HEAD-DT and C4.5. For HEAD-DT, it is actually presenting the results provided by the decision-tree algorithm tailored to the *NADH* data set and tested on the remaining five data sets. It illustrates the average accuracy and tree size according to the 10-fold cross-validation procedure. The average of the differences $\hat{\mu }$ and variance of the differences ${\hat{\sigma }}^{2}$ are also presented.

**Table 2:** Results of Experiment 1

Measure	Ligand	HEAD	C4.5	$\hat{\mu }$	${\hat{\sigma }}^{2}$	*t*
Accuracy	*ETH*	0.71 ± 0.02	0.62 ± 0.02	0.08	0.0008	6.25
	*PIF*	0.86 ± 0.00	0.80 ± 0.02	0.06	0.0004	6.22
	*TCL*	0.65 ± 0.02	0.57 ± 0.02	0.08	0.0003	9.12
	*INH*	0.84 ± 0.01	0.79 ± 0.01	0.05	0.0001	8.28
	*JPM*	0.72 ± 0.02	0.65 ± 0.02	0.07	0.0004	7.62
Tree Size	*ETH*	45.40 ± 9.88	539.80 ± 29.64	494.40	1174.04	31.48
	*PIF*	6.80 ± 0.63	283.00 ± 39.97	276.20	1603.95	15.00
	*TCL*	38.20 ± 8.17	588.00 ± 37.39	549.80	1549.73	30.40
	*INH*	12.20 ± 3.91	278.60 ± 24.80	266.40	568.71	24.31
	*JPM*	28.20 ± 6.48	483.80 ± 19.76	455.60	408.71	49.05

Table 2 presents the classification accuracy and tree size of HEAD-DT and C4.5 in the *ETH*, *PIF*, *TCL*, *INH*, and *JPM* data sets. HEAD-DT results were obtained by running a single decision-tree algorithm tailored to the *NADH* data set.

First, let us consider the accuracy results depicted in Table 2. For computing the *t* value for each data set, we have:

(3) $$t_{\text{ETH}}=\frac{0.08}{\sqrt{\left(\frac{1}{10}+\frac{1}{9}\right)\times 0.0007}}=6.25$$

(4) $$t_{\text{PIF}}=\frac{0.06}{\sqrt{\left(\frac{1}{10}+\frac{1}{9}\right)\times 0.0004}}=6.22$$

(5) $$t_{\text{TCL}}=\frac{0.08}{\sqrt{\left(\frac{1}{10}+\frac{1}{9}\right)\times 0.0003}}=9.12$$

(6) $$t_{\text{INH}}=\frac{0.05}{\sqrt{\left(\frac{1}{10}+\frac{1}{9}\right)\times 0.0001}}=8.28$$

(7) $$t_{\text{JPM}}=\frac{0.07}{\sqrt{\left(\frac{1}{10}+\frac{1}{9}\right)\times 0.0004}}=7.62$$

Regarding the *ETH* data set, the value of *t* is 6.25, and since 6.25>2.26, HEAD-DT significantly outperforms C4.5 in the *ETH* data set. The same can be said for the *PIF* (*t*=6.22), *TCL* (*t*=9.12), *INH* (*t*=8.28), and *JPM* (*t*=7.62) data sets. These results suggest that evolving a decision-tree algorithm tailored to a particular domain — recall that in this experiment, the domain was represented by the *NADH* data set — is a good idea for generating more accurate decision trees.

We can also verify in Table 2 whether the trees generated by the evolved algorithm are more comprehensible (*i.e.*, smaller) than those generated by C4.5. We can observe that HEAD-DT is able to generate much smaller trees than C4.5 for each data set. In the *ETH* data set, HEAD-DT generates trees that are, on average, 12 times smaller than those generated by C4.5. In *PIF*, this difference is even greater: HEAD-DT generates trees that are, on average, 40 times smaller than the C4.5 generated trees. The difference in the *TCL* data set is also large in favor of HEAD-DT: trees 15 times smaller, on average. The same behavior is observed in the *INH* (23 times smaller) and *JPM* (17 times smaller) data sets. These very large differences are reflected in the statistical test, as follows:

(8) $$t_{\text{ETH}}=\frac{494.4}{\sqrt{\left(\frac{1}{10}+\frac{1}{9}\right)\times 1,174.04}}=31.48$$

(9) $$t_{\text{PIF}}=\frac{276.2}{\sqrt{\left(\frac{1}{10}+\frac{1}{9}\right)\times 1,603.95}}=15.00$$

(10) $$t_{\text{TCL}}=\frac{549.8}{\sqrt{\left(\frac{1}{10}+\frac{1}{9}\right)\times 1,549.73}}=30.40$$

(11) $$t_{\text{INH}}=\frac{266.4}{\sqrt{\left(\frac{1}{10}+\frac{1}{9}\right)\times 568.71}}=24.31$$

(12) $$t_{\text{JPM}}=\frac{455.6}{\sqrt{\left(\frac{1}{10}+\frac{1}{9}\right)\times 408.71}}=49.05$$

These values indicate that HEAD-DT clearly outperforms C4.5 with statistical significance regarding tree size. The evolved algorithm that was tailored to *NADH* and then applied to the remaining three data sets is the following: 

1)Recursively split nodes with the Gain Ratio criterion;

2)Create one edge for each category in a nominal split;

3)Perform step 1 until class-homogeneity or the minimum number of 15 instances is reached;

4)Perform EBP pruning with cf = 5%;

5)When dealing with missing values:

5.1)Ignore missing values in split calculation;

5.2)Weight missing values according to the partition probability;

5.3)For classifying an instance with missing values, go to the most probable partition.

### Experiment 2 — An evolved decision-tree algorithm for each data set

In this experiment, we make use of HEAD-DT to evolve a decision-tree algorithm tailored to each ligand data set (one algorithm per data set). Table 3 presents the results of this strategy.

**Table 3:** Results of Experiment 2

Measure	Ligand	HEAD	C4.5	$\hat{\mu }$	${\hat{\sigma }}^{2}$	*t*
Accuracy	*ETH*	0.70 ± 0.02	0.62 ± 0.02	0.08	0.00074	6.28
	*PIF*	0.87 ± 0.00	0.80 ± 0.02	0.06	0.00045	6.39
	*TCL*	0.65 ± 0.02	0.57 ± 0.02	0.07	0.00022	10.85
	*NADH*	0.75 ± 0.03	0.72 ± 0.02	0.03	0.00054	2.28
	*INH*	0.83 ± 0.02	0.79 ± 0.01	0.04	0.00033	4.57
	*JPM*	0.72 ± 0.02	0.65 ± 0.02	0.07	0.00061	5.90
Tree Size	*ETH*	30.20 ± 38.09	539.80 ± 29.64	510.0	2147.38	23.95
	*PIF*	17.80 ± 13.44	283.00 ± 39.97	265.2	1174.40	16.84
	*TCL*	43.00 ± 37.49	588.00 ± 37.39	545.0	3193.11	20.99
	*NADH*	87.00 ± 36.42	360.00 ± 22.33	273.0	2379.78	12.18
	*INH*	42.80 ± 27.96	278.60 ± 24.80	235.8	1313.29	14.16
	*JPM*	121.80 ± 65.60	483.80 ± 19.76	362.0	4152.00	12.23

Table 3 presents the classification accuracy and tree size of HEAD-DT and C4.5 in the *ETH*, *PIF*, *TCL*, *NADH*, *INH*, and *JPM* data sets. HEAD-DT results were obtained by running an evolved decision-tree algorithm tailored to each data set.

Observe that HEAD-DT generates more accurate trees than C4.5 for all data sets. The values of *t* for each data set are: 6.28 (*ETH*), 6.39 (*PIF*), 10.85 (*TCL*), 2.28 (*NADH*), 4.57 (*INH*), and 5.90 (*JPM*) which means that the trees generated by the algorithms evolved by HEAD-DT outperform those by C4.5 with statistical significance in all data sets, regarding test accuracy. The next step is, once again, to verify whether the trees generated by the evolved algorithms are more comprehensible than those generated by C4.5. We can see in Table 3 that the trees generated by HEAD-DT are much smaller than those by C4.5. The values of *t* for *ETH*, *PIF*, *TCL*, *NADH*, *INH*, and *JPM* are, respectively: 23.95, 16.84, 20.99, 12.18, 14.16, and 12.23. Hence, the trees generated by HEAD-DT are significantly smaller than the trees provided by C4.5 with statistical assurance.

## Discussion

We conclude from Experiments 1 and 2 that both strategies for automatically generating decision-tree algorithms are effective for the problem of RDD with flexible-receptor docking data. Experiment 1 seems to be a better option than Experiment 2, considering that it requires a single execution of HEAD-DT, instead of multiple runs — one for each data set. After evolving a single algorithm in Experiment 1, the computational cost of applying the evolved algorithm in new data sets is very low: building a tree takes *O*(*m*×*n*log*n*) time (*m* is the number of attributes and *n* the number of instances), plus the individual complexity of a pruning method. A single execution of HEAD-DT requires operations such as breeding and fitness evaluation. Breeding takes negligible time, which is a known fact in evolutionary algorithms that deal with strings. Fitness evaluation, on the other hand, is the bottleneck of HEAD-DT, because each individual has to generate a decision tree for a given data set. We can estimate the time complexity of HEAD-DT as *O*(*i*×*g*×*m*×*n*log*n*), where *i* is the number of individuals and *g* the number of generations. In practice, however, the number of evaluations is much smaller than *i*×*g*, because repeated individuals are not re-evaluated. Also, individuals selected by elitism and reproduction are not re-evaluated, saving computational time.

Our experiments suggest that not only the resulting trees from the tailored algorithms are more accurate than those generated by C4.5, but also significantly smaller (*i.e.*, more comprehensible). The importance of generating comprehensible predictive models in several application domains has already been established. In bioinformatics, it provides advantages such as [35]: (i) improving the biologist’s confidence in the prediction; (ii) giving the biologist new insight about the data; (iii) giving the biologist ideas for hypotheses creation; and (iv) allowing the detection of errors in the model or in the data. Hence, we believe HEAD-DT is a good alternative to C4.5 for domains in which comprehensible accurate models are of great importance, such as molecular docking.

**Figure 4:**
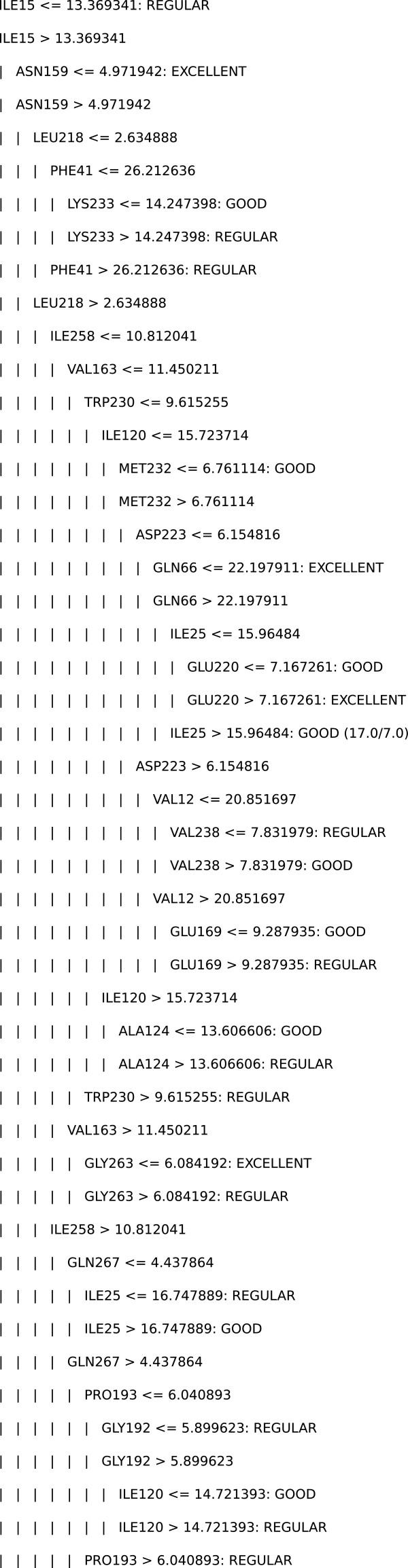
**Decision tree generated in Experiment 1 with data from the *ETH *ligand.** Figure 4 presents a decision tree generated by the algorithm evolved in Experiment 1 (tailored to the *NADH* data set), which was trained with data from the *ETH* ligand.

Considering the importance of model-comprehensibility to confirm or reject hypotheses regarding the available data, we briefly discuss some intriguing facts regarding the decision tree generated in Experiment 1 with data from the *ETH* ligand. The generated tree has a total of 49 nodes and 25 leaves (see Figure 4). We first highlight that only the three levels of FEB that indicate a reasonable conformation of *InhA* to *ETH* (REGULAR, GOOD, EXCELLENT) appear in the leaves. More specifically, the decision tree ignored rules that could predict conformations that are irrelevant with respect to the docking experiments.

Regarding our findings within the decision tree model, note that *ETH* binds to *InhA* as an adduct (*ETH-NADH*) formed with the *NADH* coenzyme [56]. The active site of the *InhA* receptor, composed of the coenzyme and substrate-binding cavity, is almost completely filled during *InhAETH-NADH* interaction [13]. Almost all residues found in the *ETH* decision tree model are directly related or very close to the definition of the receptor active site. For instance, we verify that three of them (PHE41, GLY192, PRO193) are listed as the top-25 most significant residues to *InhA* flexibility, as reported in [53]. PHE41 helps fit the adenine portion of *NADH* into its binding site while GLY192 and PRO193 are located in the substrate-binding loop region. This is a remarkable prediction since these residues do actually make direct contact with the adduct in the crystal structure [13], meaning that they are determinant to the inhibition of *InhA* by *ETH*. In addition, of other six residues that appear in the model (VAL12, ILE15, ILE120, ALA124, ASN159, VAL163), one (ILE15) interacts directly with the adduct while the others are structural nearest-neighbors to the residues that belong to the top-25. Having such information at hand allowed the biologist to improve his/her understanding regarding *InhA* flexibility and function, as well as its behavior when docking with *ETH* and other similar ligands.

Looking at the leaves that classify snapshots as EXCELLENT, we noticed that the residues in their paths are quite far from the receptor binding pocket. Even though these residues seem to be irrelevant at first sight, they are actually determining whether the residues that are closer to the receptor can actually provide lower FEB values, and thus better conformation of *InhA* to *ETH*. For instance, we obtained the following rules in the decision tree model: 

(1)IF (ILE15 > 13.37) AND (ASN159 <= 4.97)

THEN FEB = EXCELLENT

(2)IF (ILE15 > 13.37) AND (ASN159 > 4.97) AND (LEU218 > 2.63) AND (ILE258 <= 10.81) AND (VAL163 > 11.45) AND (GLY263 <= 6.08)

THEN FEB = EXCELLENT.

Rule (1) indicates that an excellent conformation of *InhA* to *ETH* imply in the receptor residue *Isoleucine-15* being far from the binding pocket (in distances greater than 13.37 Å) and, at the same time, the residue *Asparagine-159* being relatively close to the receptor (in distances lesser than 4.97 Å). Inspection of the *InhA* - *ETH-NADH* crystal structure [13] confirms this finding.

**Figure 5:**
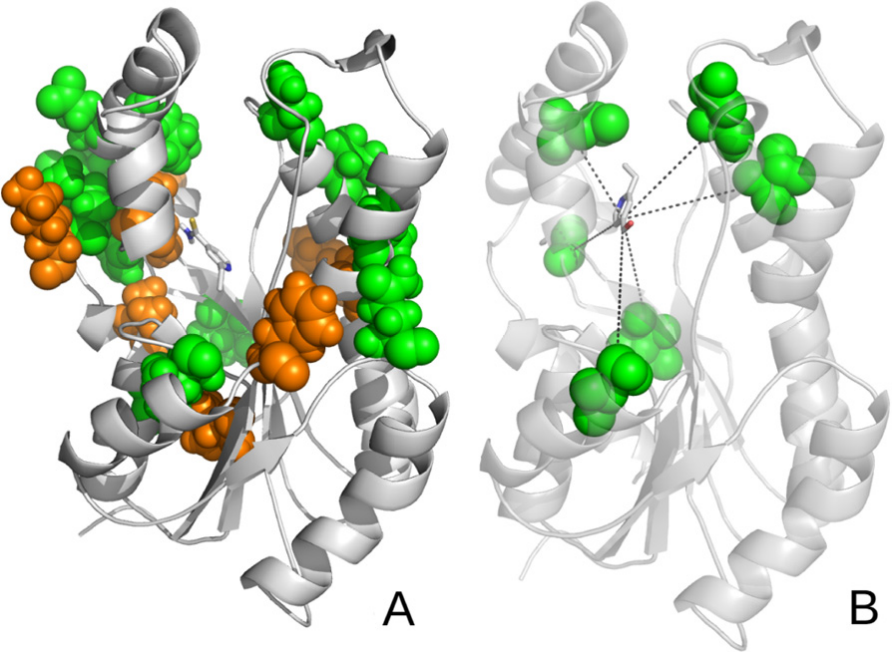
**Residue-attribute mapping for the *ETH *decision tree.** Figure 5 is composed as follows: (**A**) The main chain of the *FFR-InhA* receptor is represented by grey ribbons. The amino acids residues identified by the decision tree are represented as space-filled atoms. For clarity, only the residues classified as Excellent (green) and Good (orange) are shown. (**B**) The main chain of the experimental structure (PDB ID: 2HI9) is shown in transparent grey ribbons together with the mapped residues of rule (2) (Excellent) of the decision tree and their distances (in Å) to the ligand. The *ETH* ligand is represented by stick models (carbon: grey; nitrogen: blue; oxygen: red; sulphur: yellow).

Conversely, rule (2) indicates that a conformation of *InhA* to *ETH* may also be excellent if residue *Asparagine-159* is far from the receptor (a distance greater than 4.97 Å), though assuming that the remaining residues presented in rule (2) meet their distance requirements. Figure 5 shows the residue-attribute mapping for the *ETH* ligand. In general the agreement between predicted and experimental binding modes is very satisfactory, as illustrated by the mapping of rule (2) to the experimental structure (Figure 5B). Only two residues, ILE258 and GLY263, did not satisfy the rule. These exceptions can be explained by the fact that the rules are based on a fully-flexible (FFR) model of the *InhA* receptor. We believe that structural differences between snapshots in the FFR model create a space in the receptor’s binding cavities, which differs from the one we see in the rigid, crystal structure. The FFR model of *InhA* can therefore accommodate a more diverse range of ligand conformations and orientations.

All the information presented so far is exactly what we expect from a comprehensible model generated from the flexible-receptor molecular docking data: helping to significantly reduce the number of snapshots in future docking experiments. Hence the importance of providing comprehensible models, like those produced by decision trees and decision rules. At the same time, it must be pointed out that very large decision trees (such as those induced by C4.5) are not of easy interpretation, since the specialist would need several hours to visually inspect interesting rules in a 500-node decision tree. Whenever two decision tree models of similar performance are compared, one should prefer the smaller one, as stated by the *Occam’s Razor* principle. Recall that HEAD-DT evolved algorithms that were able to induce significantly more accurate and comprehensible decision trees than C4.5.

## Conclusions

In this work, we proposed a hyper-heuristic algorithm capable of automatically designing decision-tree induction algorithms tailored to specific domains, namely HEAD-DT. We investigated its efficiency in the prediction of the free energy of binding of a drug candidate with enzyme *InhA* associated with *Mycobacterium tuberculosis*. More specifically, we performed several experiments with flexible-receptor docking data for rational drug design, a bioinformatics application of known importance.

We compared the algorithms automatically designed by HEAD-DT to traditional state-of-the-art decision-tree induction algorithm C4.5 [20]. We assessed the performance of HEAD-DT through two different measures: accuracy and tree size. The experimental analysis suggested that HEAD-DT can generate algorithms which perform significantly better than C4.5. These algorithms can also induce significantly smaller trees, regarding the domain of flexible-receptor docking data. The resulting decision trees were analyzed by a biologist, who was able to extract several interesting rules for helping future docking experiments. For instance, the biologist verified that three residues selected by the decision tree model (PHE41, GLY192, PRO193) are determinant to the inhibition of *InhA* by *ETH*.

We believe that these results indicate that HEAD-DT can be an effective algorithm for domain-specific applications of decision trees, specially bioinformatics. As future work, we plan to investigate whether a more sophisticated search system, such as grammar-based genetic programming, can outperform our current HEAD-DT implementation. We intend to employ HEAD-DT in further experiments of flexible-receptor molecular docking data, for helping in the discovery of new drug candidates to *Mycobacterium tuberculosis*. In addition, considering the good performance of HEAD-DT over flexible-receptor docking data, we intend to test it in other relevant bioinformatics problems.

## Competing interests

The authors declare that they have no competing interests.

## Authors’ contributions

RB implemented the method and wrote the manuscript. RB, MB, and AC designed the experiments, evaluated the experimental results, and assessed their statistical significance. AW, KM, DR, and ONS generated and preprocessed the biological data, and also interpreted the resulting decision trees. ONS interpreted and validated the biological findings. All authors read and approved the final manuscript.
